# Supplementary material for: Design, Synthesis, and Antiproliferative Activity of Selective Histone Deacetylases 6 Inhibitors Containing a Tetrahydropyridopyrimidine Scaffold
Source: Molecules. 2023 Oct 29;28(21):7323. doi: 10.3390/molecules28217323 (PMC10648541; doi:10.3390/molecules28217323)

Supporting Information for

Design, Synthesis, and Antiproliferative Activity of Selective Histone Deacetylases 6 Inhibitors Containing a Tetrahydropyridopyrimidine Scaffold

^{1}H-NMR, ^{13}C-NMR and HR-MS spectras of synthesized compounds of **8a–h（Page1-8）**

8-(2, 4-diphenyl-5, 8-dihydropyrido[3, 4-*d*]pyrimidin-7(6*H*)-yl)-*N*-hydroxy-8-oxooctanamide (**8a**)

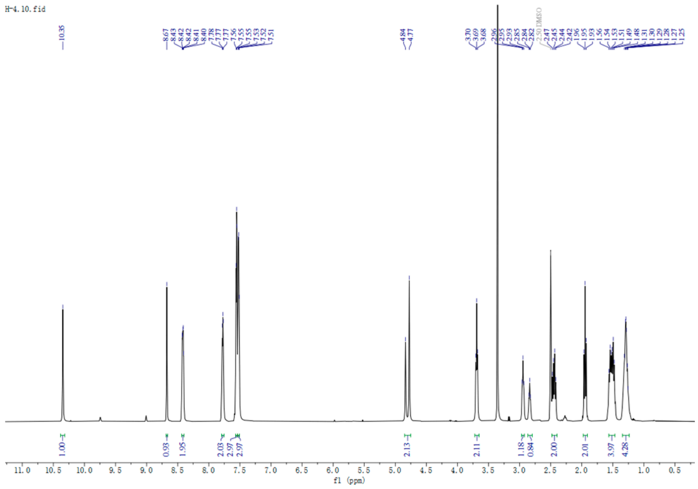

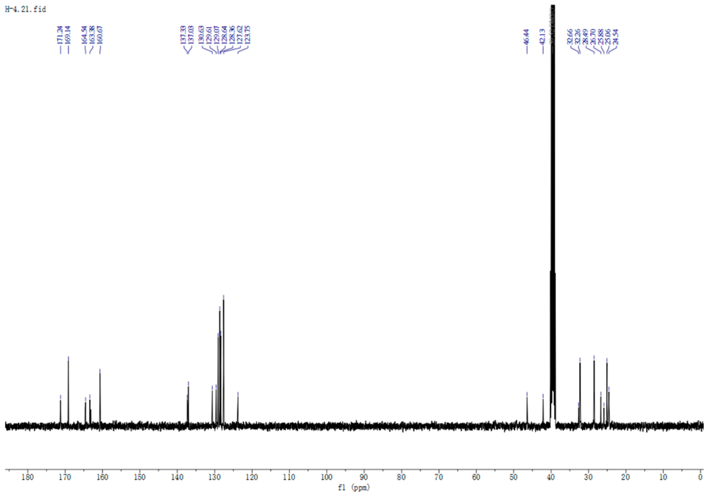

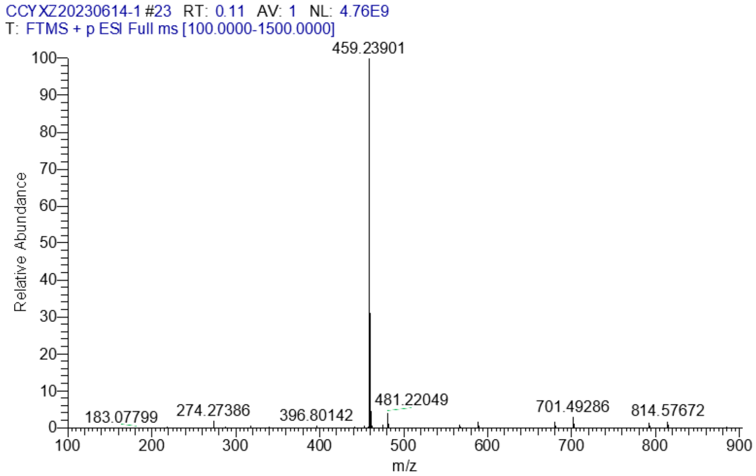

8-(2, 4-di-p-tolyl-5, 8-dihydropyrido[3, 4-*d*]pyrimidin-7(6*H*)-yl)-*N*-hydroxy-8-oxooctanamide (**8b**)

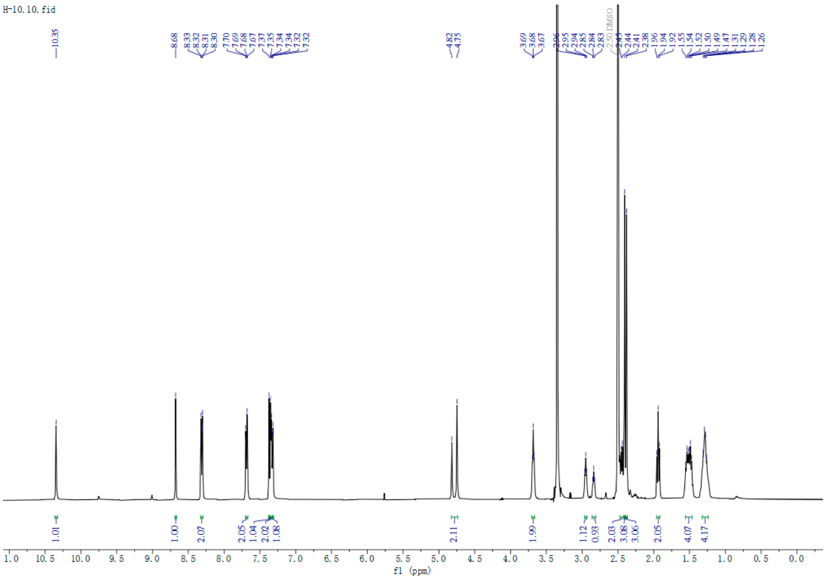

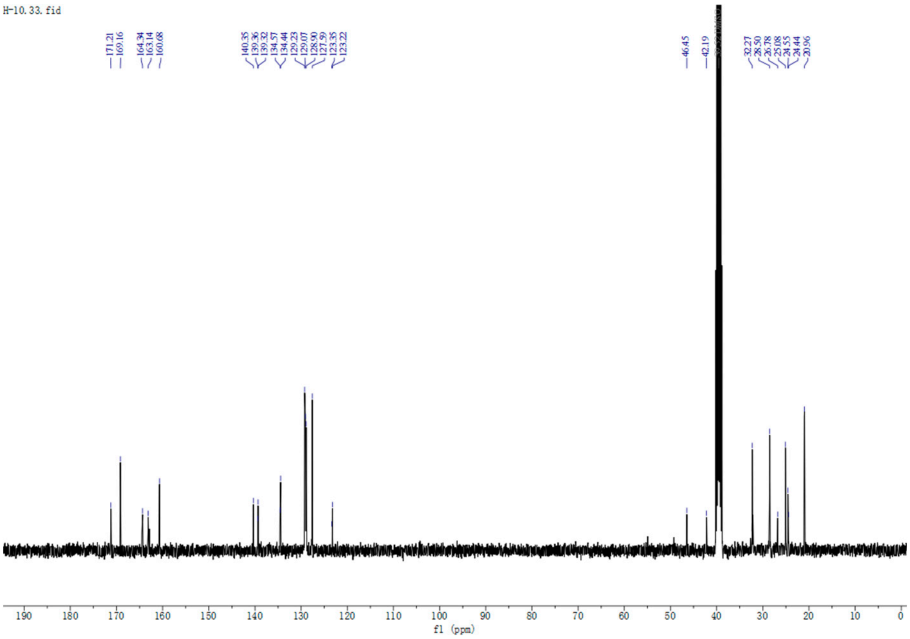

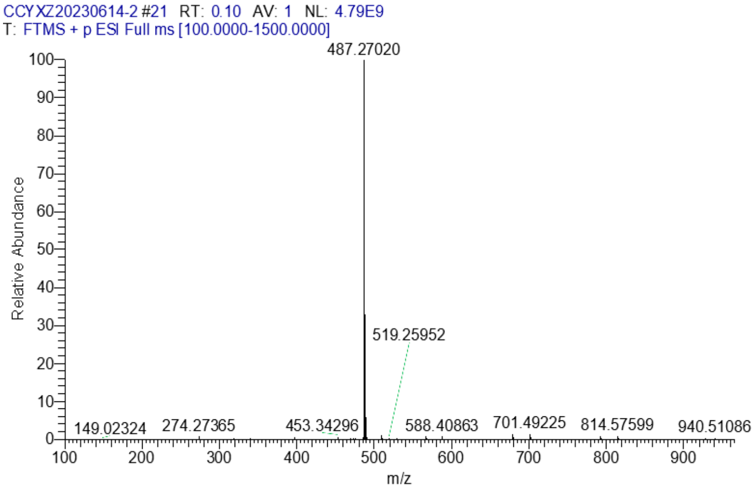

8-(2, 4-bis(4-methoxyphenyl)-5, 8-dihydropyrido[3, 4-*d*]pyrimidin-7(6*H*)-yl)-*N*-hydroxy-8-oxooctanamide (**8c**)

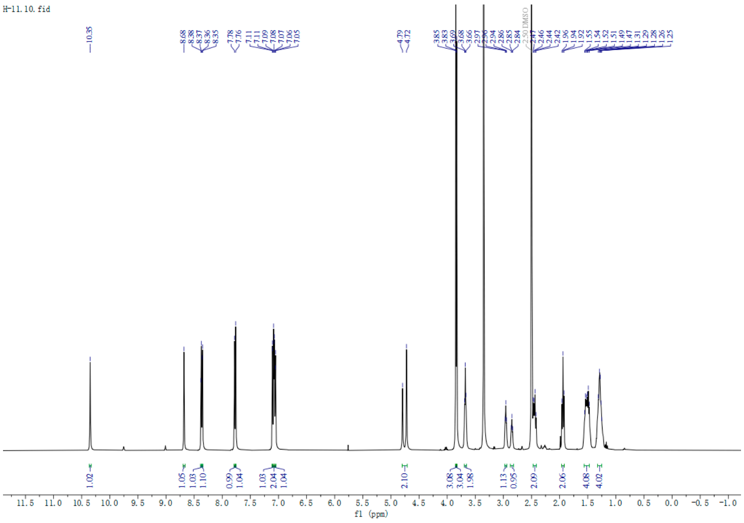

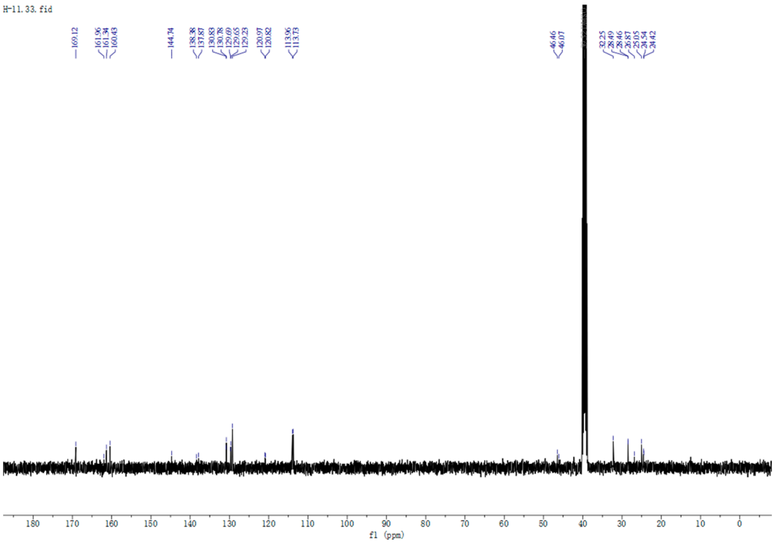

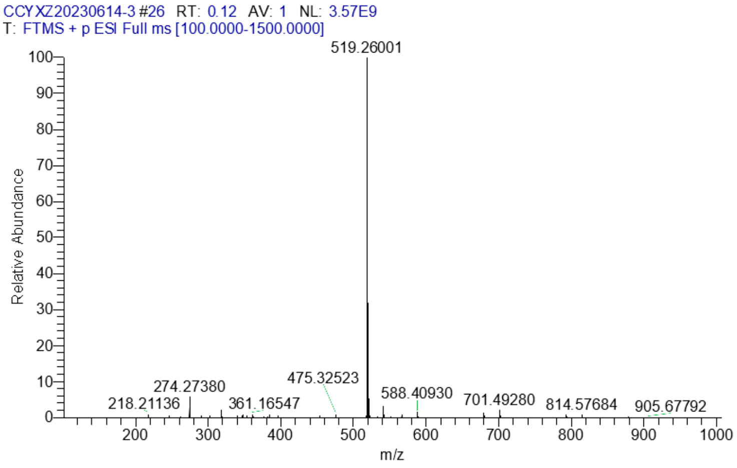

8-(2, 4-di(furan-3-yl)-5, 8-dihydropyrido[3, 4-*d*]pyrimidin-7(6*H*)-yl)-*N*-hydroxy-8-oxooctanamide (**8d**)

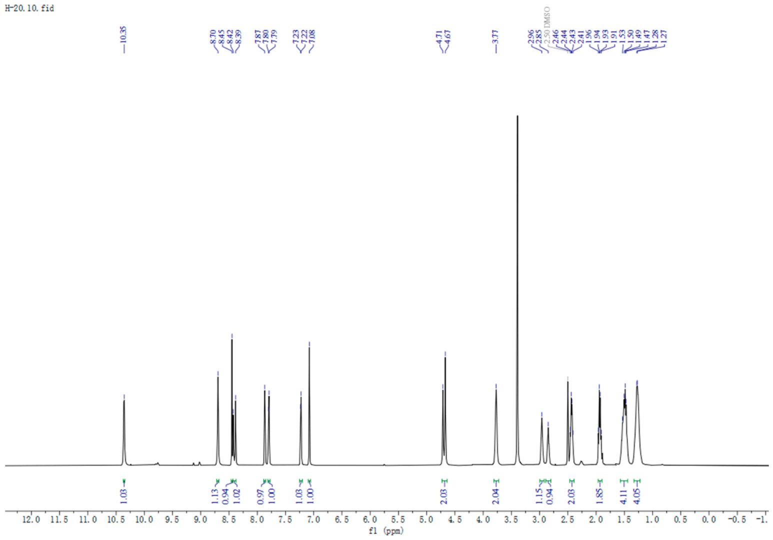

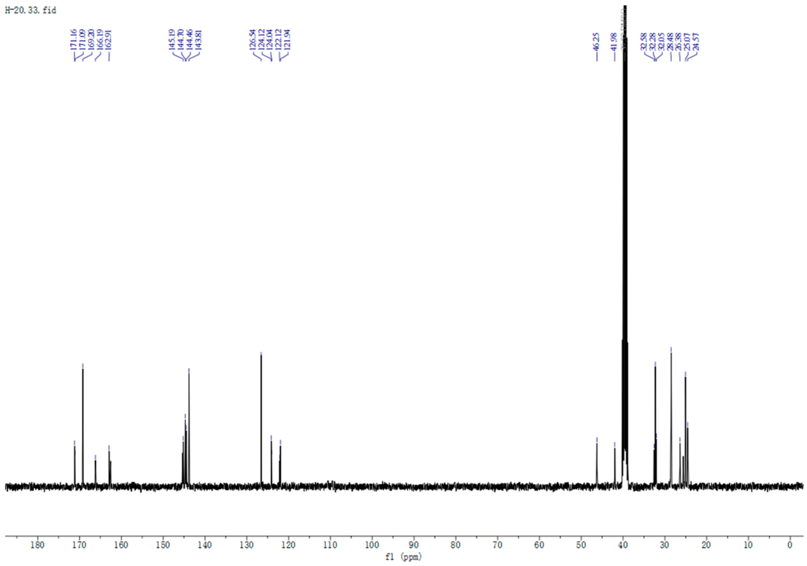

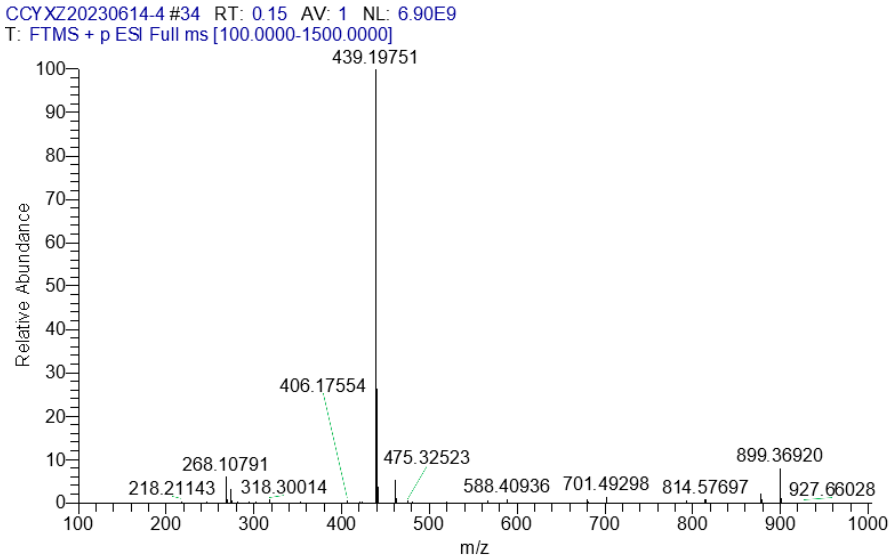

8-(2, 4-di(thiophen-3-yl)-5,8-dihydropyrido[3, 4-*d*]pyrimidin-7(6*H*)-yl)-*N*-hydroxy-8-oxooctanamide (**8e**)

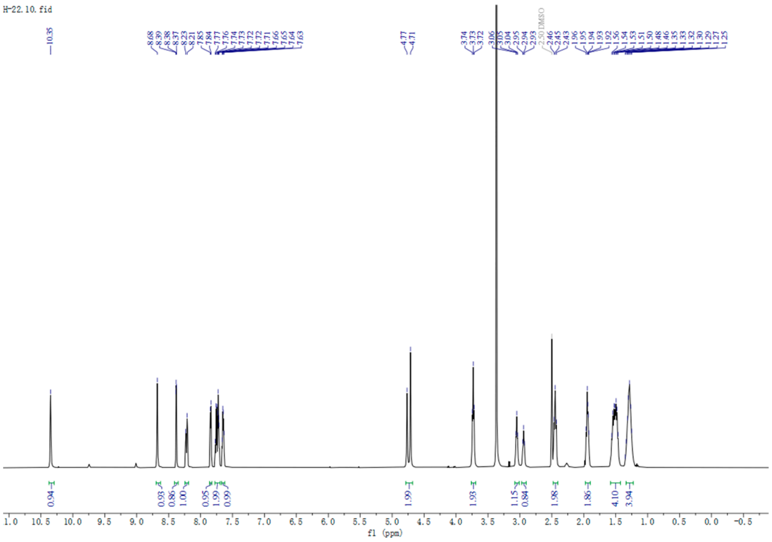

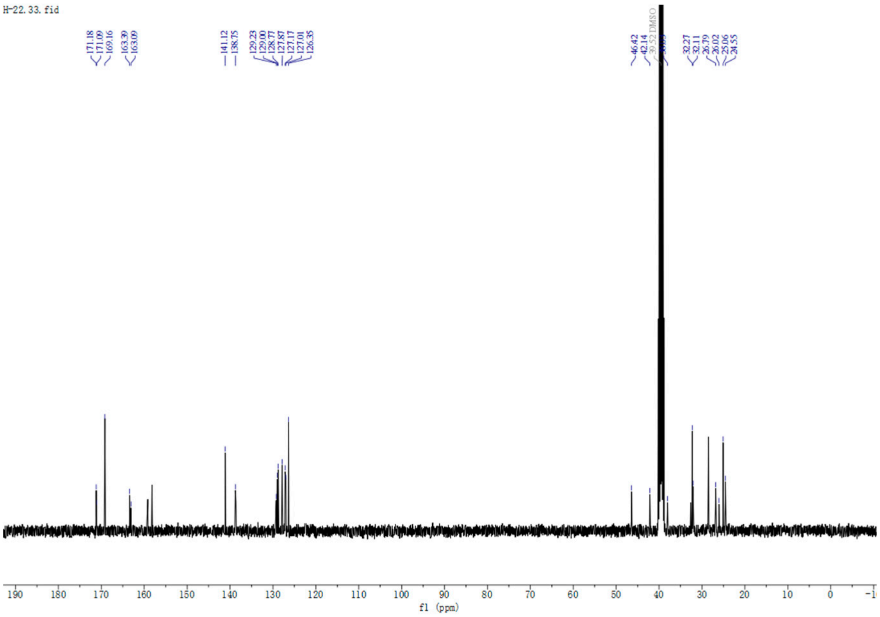

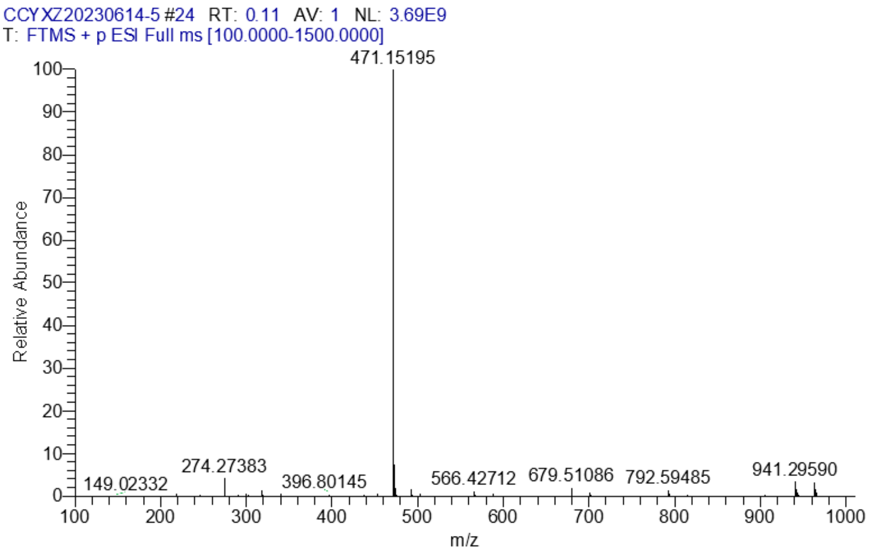

8-(2, 4-bis(3-methoxyphenyl)-5, 8-dihydropyrido[3, 4-*d*]pyrimidin-7(6*H*)-yl)-*N*-hydroxy-8-oxooctanamide (**8f**)

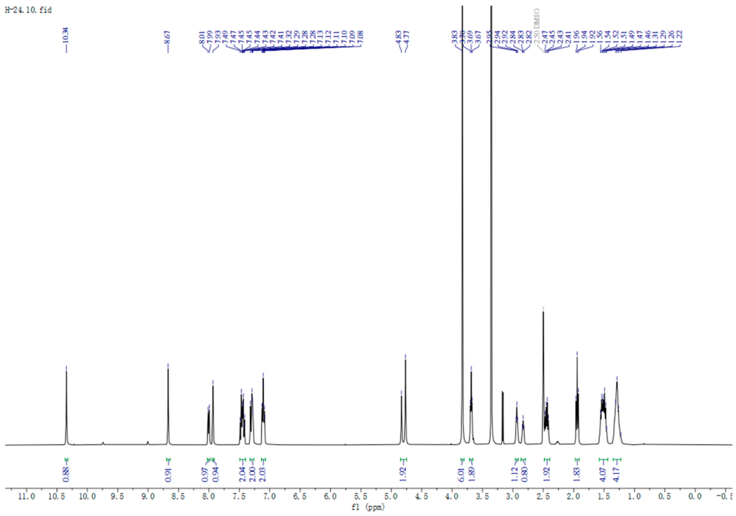

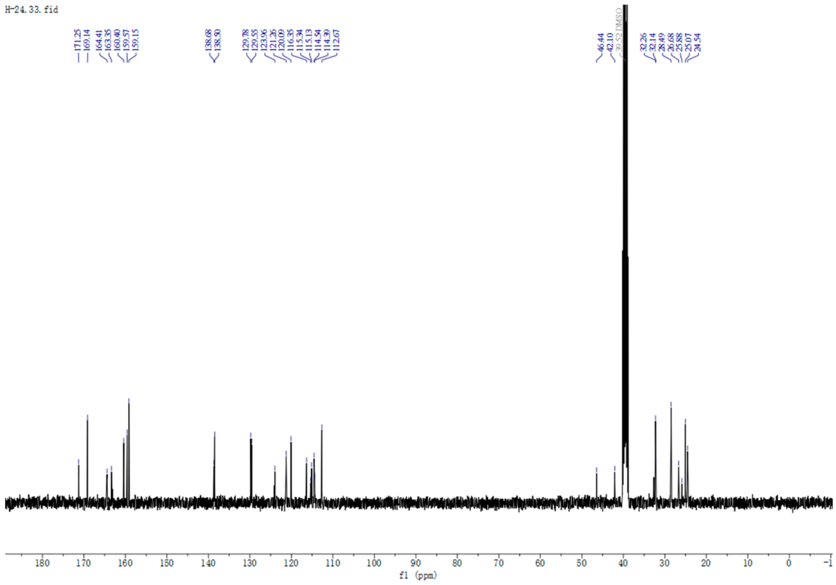

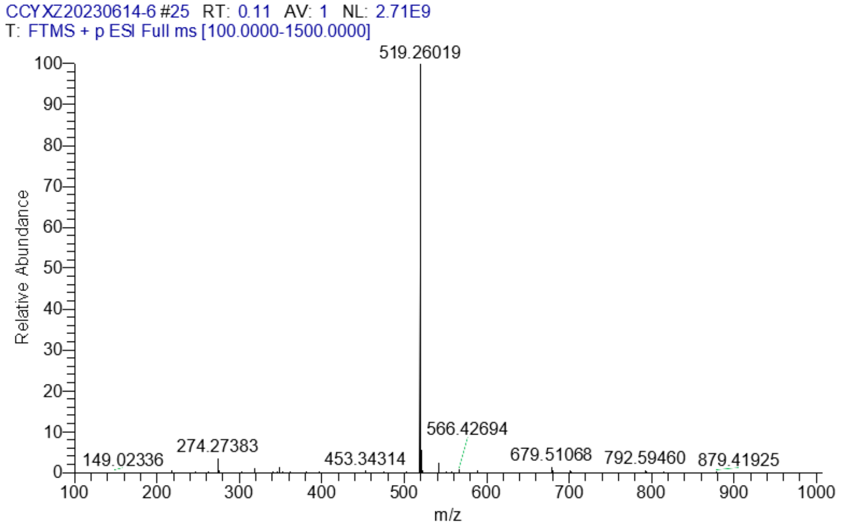

8-(2, 4-di(thiophen-2-yl)-5, 8-dihydropyrido[3, 4-d]pyrimidin-7(6*H*)-yl)-*N*-hydroxy-8-oxooctanamide (**8g**)

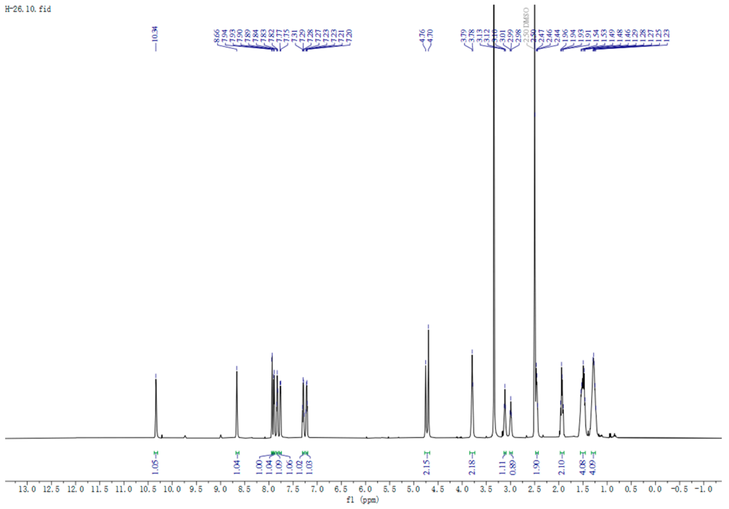

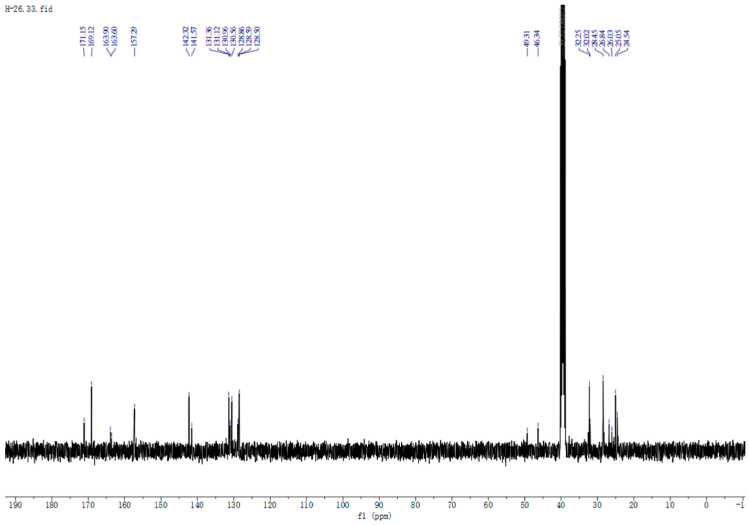

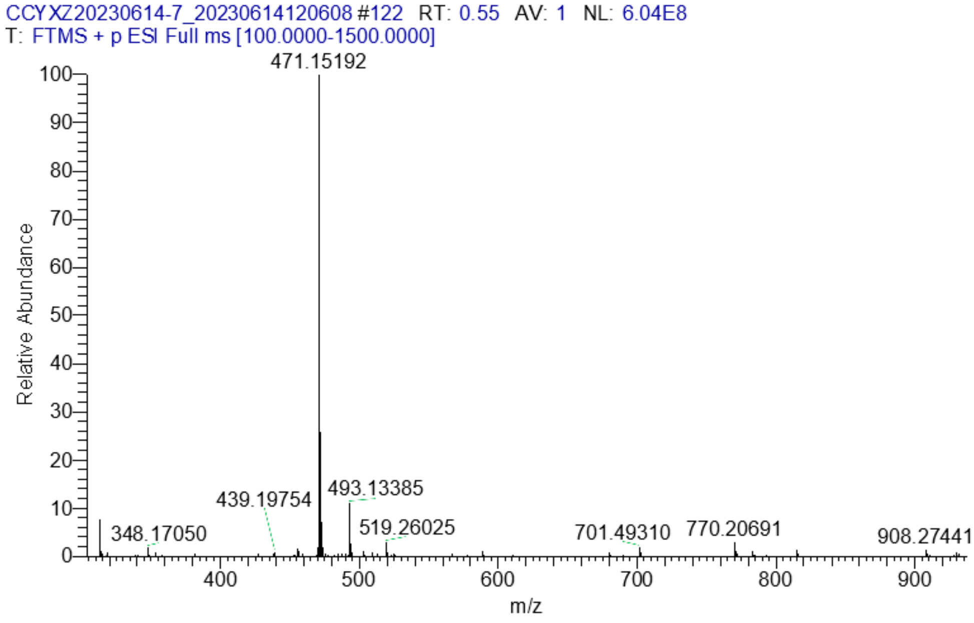

8-(2, 4-bis(4-(trifluoromethyl)phenyl)-5, 8-dihydropyrido[3, 4-*d*]pyrimidin-7(6*H*)-yl)-*N*-hydroxy-8-oxooctanamide (**8h**)

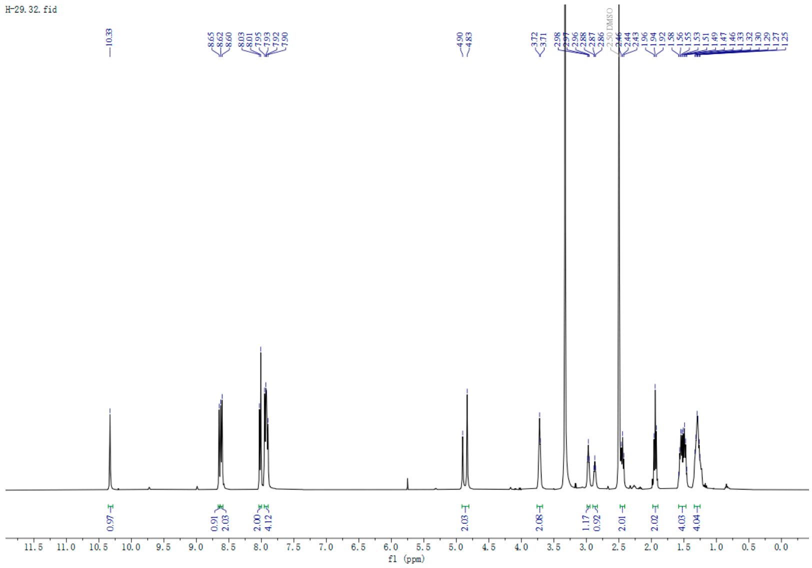

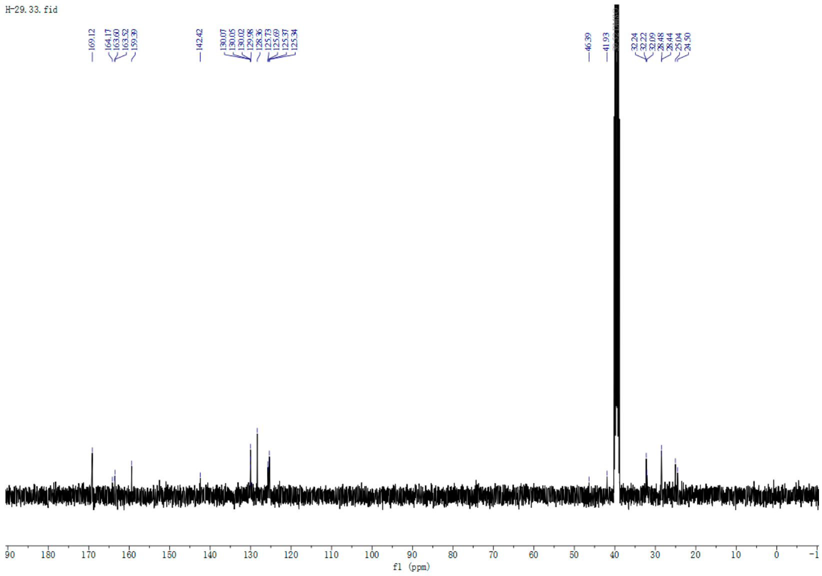

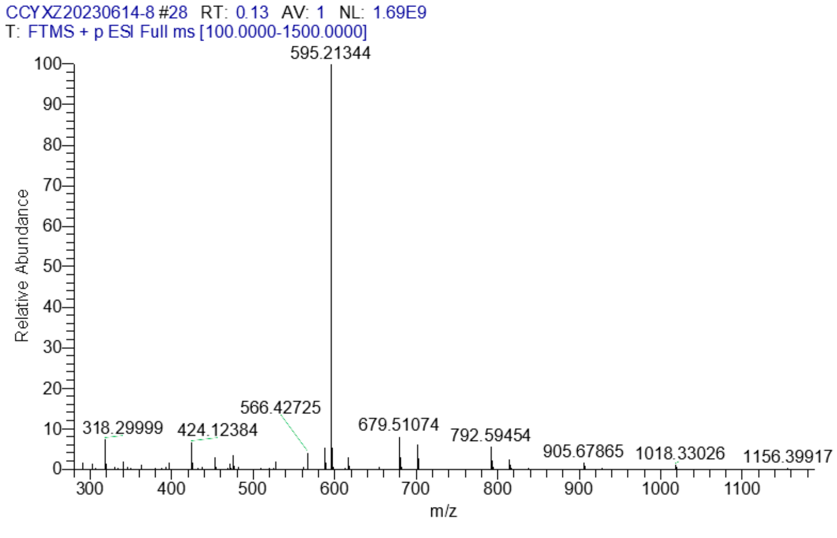

Growth percents of **8f** against 59 tumor cells in NCI-60 program

Developmental Therapeutics Program One Dose Mean Graph	NSC: D-835719 / 1	Conc: 1.00E-5 Molar	Test Date: Feb 28, 2022
	Experiment ID: 2202OS75		Report Date: Sep 14, 2023

Panel/Cell Line	Growth Percent
Leukemia	
CCRF-CEM	15.02
HL-60(TB)	-4.14
K-562	9.84
MOLT-4	12.33
RPMI-8226	11.27
SR	15.70
Non-Small Cell Lung Cancer	
A549/ATCC	12.28
EKVX	15.61
HOP-62	0.93
HOP-92	-35.56
NCI-H226	6.26
NCI-H23	-48.99
NCI-H322M	10.98
NCI-H460	4.48
NCI-H522	-30.90
Colon Cancer	
COLO 205	-80.44
HCC-2998	-63.91
HCT-116	4.00
HCT-15	-6.55
HT29	4.73
KM12	11.56
SW-620	-58.20
CNS Cancer	
SF-268	7.80
SF-295	-49.00
SF-539	1.02
SNB-19	-8.90
SNB-75	29.00
U251	-37.72
Melanoma	
LOX IMVI	-96.92
MALME-3M	-34.36
M14	9.54
MDA-MB-435	4.16
SK-MEL-2	-20.43
SK-MEL-28	-2.70
SK-MEL-5	-87.82
UACC-257	1.34
UACC-62	-87.20
Ovarian Cancer	
IGROV1	-46.31
OVCAR-3	-11.25
OVCAR-4	32.93
OVCAR-5	-22.41
OVCAR-8	6.42
NCI/ADR-RES	-44.45
SK-OV-3	-39.05
Renal Cancer	
786-0	10.91
A498	-42.37
ACHN	-74.19
CAKI-1	-4.81
SN12C	-0.36
TK-10	-17.20
UO-31	-18.83
Prostate Cancer	
PC-3	16.71
DU-145	10.18
Breast Cancer	
MCF7	7.92
MDA-MB-231/ATCC	-21.81
HS 578T	2.21
BT-549	18.10
T-47D	-13.06
MDA-MB-468	-0.98
Mean	-13.86
Delta	83.06
Range	129.85

Mean Growth Percent - Growth Percent

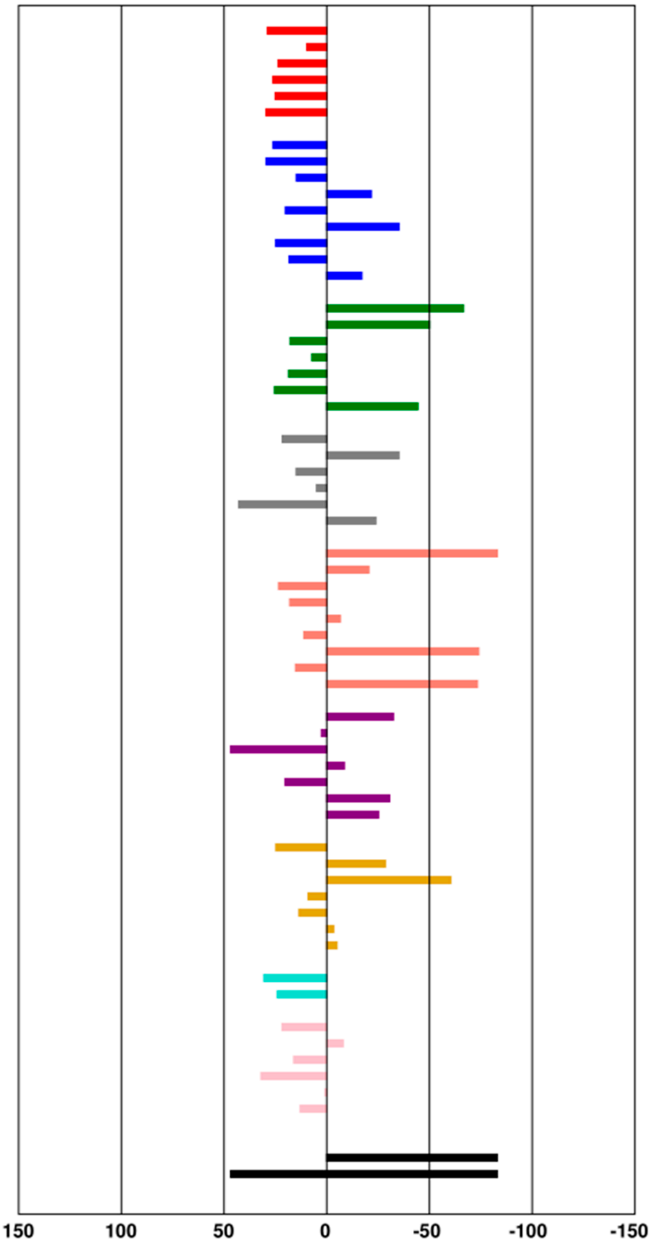

Supplement: Supplementary file 1 [file molecules-28-07323-s001.zip › molecules-2648315-supplementary.pdf]
